# An Update on Advancements and Challenges in Inhalational Drug Delivery for Pulmonary Arterial Hypertension

**DOI:** 10.3390/molecules27113490

**Published:** 2022-05-29

**Authors:** Vinit Agnihotri, Yogeeta Agrawal, Sameer Goyal, Charu Sharma, Shreesh Ojha

**Affiliations:** 1 Shri Vile Parle Kelavani Mandal’s Institute of Pharmacy, Dhule 424 001, India; vinit.agnihotri@svkm.ac.in (V.A.); goyal.aiims@gmail.com (S.G.); 2 Department of Internal Medicine, College of Medicine and Health Sciences, United Arab Emirates University, Al Ain P.O. Box 15551, United Arab Emirates; charusharma@uaeu.ac.ae; 3 Department of Pharmacology and Therapeutics, College of Medicine and Health Sciences, United Arab Emirates University, Al Ain P.O. Box 15551, United Arab Emirates

**Keywords:** particles deposition, inhaled drug delivery, lipid nanoparticles, pulmonary arterial hypertension, targeted delivery, endothelial dysfunction

## Abstract

A lethal condition at the arterial–alveolar juncture caused the exhaustive remodeling of pulmonary arterioles and persistent vasoconstriction, followed by a cumulative augmentation of resistance at the pulmonary vascular and, consequently, right-heart collapse. The selective dilation of the pulmonary endothelium and remodeled vasculature can be achieved by using targeted drug delivery in PAH. Although 12 therapeutics were approved by the FDA for PAH, because of traditional non-specific targeting, they suffered from inconsistent drug release. Despite available inhalation delivery platforms, drug particle deposition into the microenvironment of the pulmonary vasculature and the consequent efficacy of molecules are influenced by pathophysiological conditions, the characteristics of aerosolized mist, and formulations. Uncertainty exists in peripheral hemodynamics outside the pulmonary vasculature and extra-pulmonary side effects, which may be further exacerbated by underlying disease states. The speedy improvement of arterial pressure is possible via the inhalation route because it has direct access to pulmonary arterioles. Additionally, closed particle deposition and accumulation in diseased tissues benefit the restoration of remolded arterioles by reducing fallacious drug deposition in other organs. This review is designed to decipher the pathological changes that should be taken into account when targeting the underlying pulmonary endothelial vasculature, especially with regard to inhaled particle deposition in the alveolar vasculature and characteristic formulations.

## 1. Introduction

Cardiopulmonary disarray of arterial pressure that initiates a series of lethal events at the juxtaposition of capillaries and alveoli leads to pulmonary arterial hypertension (PAH). The characteristic vascular remodeling of distal arteries at the alveolus is followed by increased pressure of the arterioles above 25 mmHg, an ailment that is fatal and complex for the vasculature in the lungs, i.e., pulmonary circulation. This increased flow resistance in PAH, as well as the resulting overload at RV [1], causes diastolic dysfunction, which drives the cardiopulmonary vasculature to fibrosis, hypertrophy, and hyperplasia [2,3]. All the above episodes lead to failure of the right heart, which is a significant provenance of mortality in PAH patients [4]. Hemodynamically, there is an elevation in mean pulmonary artery pressure (mPAP) ≥ 30 mmHg, while standard left ventricular refilling pressures are retained at normal levels. Nevertheless, the mPAP at rest is ≥25 mmHg [5].

Therapeutics for PAH operate in the pursuit of chief pathways such as inhaled NO or Nitric Oxide/cGMP, prostacyclin and its analogs, the endothelin pathway, soluble guanylate cyclase modulators, and/or phosphodiesterase-5 inhibitors [6]. Novel molecular pathways/targets include endothelial progenitor cells, tyrosine kinase pathway, RhoA/Rho kinase, Vasoactive intestinal peptide (VIP) pathway, and miRNA in PAH therapeutics. Because of its higher delivery capabilities, including delivering some moderately low-dose molecules to deep lungs, drug targeting to the pulmonary alveolus has been the primary route. Likewise, it also has an advantage where systemic side effects need to be kept at a minimum and a faster onset of action is required [7]. A skinny epithelium of the pulmonary tract provides a higher surface area that is crucial for targeting such tiny destinations [8]. However, the successful dispersion of powder to generate an effective aerosol mist suitable for inhalation is equally important. The molecule has to bear the preformulation stress via freeze-drying, milling, and/or spray-drying. Additionally, the surface traits of the powder formulation are of great importance since many characteristics of dosage form will influence the number of drugs that is essentially available to the arterial–alveolar juncture. For instance, the de-agglomeration of particles, inter and intra particular formation of cluster bridges, etc., may lead to on-time changes in particle size. This ultimately leads to particle dispersion variations in an aerosol mist. However, despite the newer therapeutic targets being investigated, there is a stringent need for the molecule to reach the desired site of the pulmonary vasculature in PAH. Additionally, the pulmonary route has certain shortcomings associated with respiratory barriers, which may hamper drug retention and, therefore, drug availability in the lungs [9,10,11]. Although the inhalation route may help one way or another, the short biological half-life and off-target side effects of drug molecules create a need for the development of new carriers that are efficient at site-specific targeting at pulmonary locations in PAH.

In this review, we have focused on reviewing pathological challenges for targeting pulmonary arterial hypertension and analyzing the formulation of an aerosol mist. Additionally, we attempted to highlight the development of characteristics of inhalation carriers for site-specific targeting of drugs in pulmonary arterial hypertension.

## 2. The Advantages of Inhaled Medication Delivery

The inhalation route has several appealing properties for treating pulmonary hypertension, including direct drug delivery to the target organ, which improves pulmonary specificity and reduces systemic side effects. It can also improve ventilation/perfusion matching by dilation of arteries supplying ventilated areas, allowing for improved gas exchange.

## 3. Adverse Functional Changes Associated with PAH

**Figure 1:**
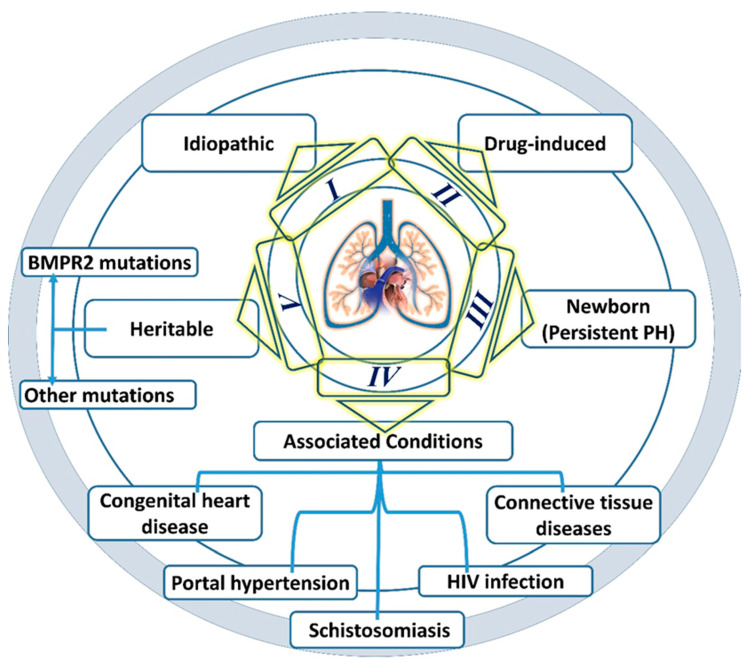
Classification of pulmonary arterial hypertension.

A cluster of clinical symptoms marked by persistent vasoconstriction of small pulmonary arteries and increased impendence in pulmonary circulation. An active manifestation of PAH triggers a series of events via the occlusion of the pulmonary vessel and augmented the vascular remodeling of pulmonary arterioles, which eventually leads to mPAP exceeding 25 mmHg at rest. Principally, PAH is grouped into I to V classes subject to associated comorbidities [2,12]. The detailed graphical representation of PAH classes is shown in Figure 1. Hemodynamically, 15 mmHg pre-capillary pressure, i.e., pulmonary artery wedge pressure and more than 3 Wood units of pulmonary vascular resistance (PVR) confer the foundation of PAH. The hemodynamic of high pressure provokes endothelial dysfunction and injury to pulmonary vasculature [13].

**Figure 2:**
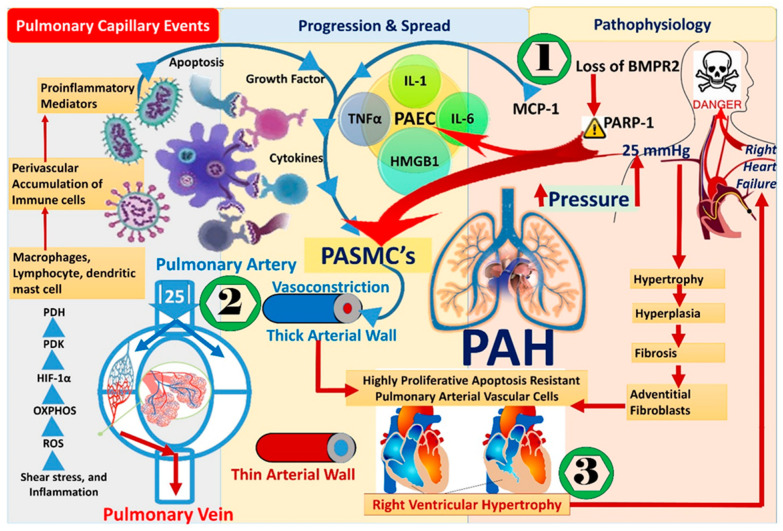
Pathological hallmarks of pulmonary arterial hypertension including events at capillary level, progression, and spread. (1) Events at the genetic level, (2) events at pulmonary artery smooth muscle cells. (3) Events leading to right heart failure (blue arrows represents molecular pathogenesis, and red arrows represents cellular impairment).

The abovementioned episodes of PA endothelial cell (PAEC) dysfunction are characteristic materials that are vital to the progression of PAH [14]. The pathological basis of PAEC may vary from epigenetics to shear stress, oxidative stress, and inflammation [15]. Moreover, PAECs that undergo apoptosis result in the perivascular accumulation of immune cells that release growth factors and activate a variety of pro-inflammatory cytokines, particularly tumor necrosis factor (TNFα) and interleukin-1 (IL-1), interleukin-6 (IL-6) (Figure 2). The cells producing proinflammatory mediators (for example, dendritic cells, lymphocytes and macrophages, and mast cells) and malfunctioned endothelial cells cumulatively liberate the nuclear protein, i.e., high mobility group-box (HMGB1), which further aggravates vessel constrictions and inflammatory responses to vascular impairment. Reports suggest that the release of HMGB1 is related to the manifestation and progression of PAH. The intimal hypertrophies at pulmo-vascular sites due to chemotaxis of the smooth muscle cells are consequences provoked by HMGB1. The associated transformation leads to a progressive tapering of the vascular lumen and likely boosts a widespread occlusion of pulmonary arterioles. 

The above course of events is attributed to the appearance of PA vascular cells that are resistant to apoptosis and are extremely proliferating. This constitutes an impairment of adventitial fibroblasts, PA smooth muscle cells (PASMCs), and PAECs. As a consequence of vasoconstriction at the pulmonary artery and the progression of disease, the arterial wall becomes thick. In addition, left-to-right switching of congenital heart defects results from an increased afterload of RV and can lead to hyperkinetic PAH followed by heart failure and premature death [14,15,16,17,18,19,20]. Furthermore, genetic damage to bone morphogenetic protein receptor (BMPR2) signaling or hypoxia [21,22] in PAEC dysfunction may provoke DNA damage. In addition, MCP-1 (monocyte/macrophage chemoattractant protein-1), a powerful chemokine and mononuclear cell activator, may have a role in the early onset and/or development of pulmonary hypertension (PH). The overexpression of Poly (ADP)-ribose-1 (PARP-1), a component/marker of the DNA repair/damage, designates pulmonary artery smooth muscle cells (PAH-PASMCs) in pulmonary hypertension [23]. The intensified proliferation of PAH-PASMCs results in the generation of reactive oxygen species (ROS) is caused by mitochondrial dysfunction. ROS further exacerbates angiogenesis marked by intracellular Ca^2+^ influx and the subsequent remodeling of the pulmonary vasculature [13,24,25,26,27]. These metabolic transformations promote oxidative phosphorylation (OXPHOS) and glycolysis [28]. At the transcriptional level, the arousal of the transcription factor, Hypoxia-inducible factor 1-alpha (HIF-1α), declines pyruvate metabolism via the augmentation of pyruvate dehydrogenase kinase (PDK) expression and the deactivation of the pyruvate dehydrogenase (PDH). All these molecular events induce increased spread and endurance of PAH cells. Furthermore, alterations in mitochondrial protein profiles, mitochondrial shape, and plug of Heat Shock Protein-90 accumulated in the mitochondria of PAH-PASMCs; they grant PAH cell survival [22].

Endothelial–mesenchymal transition (EndMT) is another prominent player in vascular remodeling and vascular lumen thickening in PAH that is recognized by the accumulation of mesenchymal cells transformed from PAECs. These cellular events exacerbate PAH progression through PASMCs/fibroblasts (Figure 2) [22,29,30].

**Figure 3:**
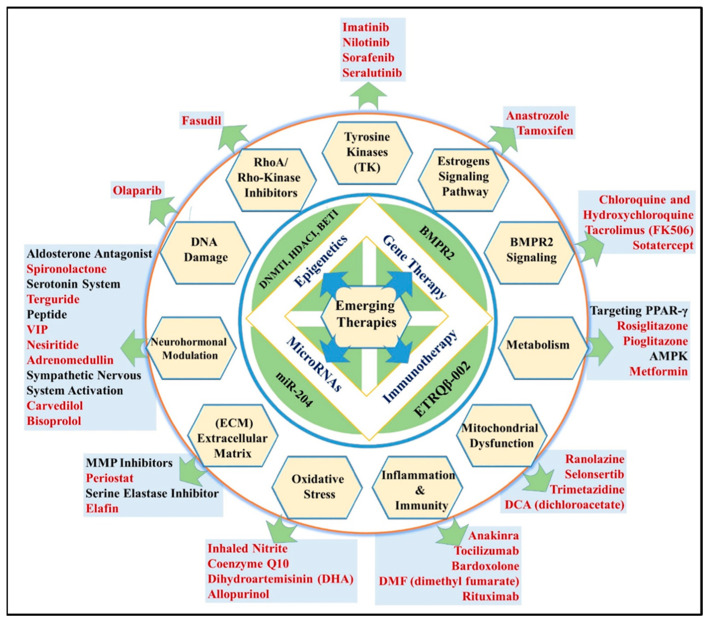
Molecules and pharmacological targets implicated in PAH (black fonts represent category of drugs; red fonts represent drug).

The pathology of PAH is very vast, and discussions of all particulars are beyond the scope of this report. In sum, the pathophysiology of PAH could be assorted by virtue of inflammation, growth factors, metabolic abnormalities, mitochondrial dysfunction, transcription factors, and growth factor-dependent sustained proliferation of PAH. The alveolar surface is overlaid by AEC I and ACE II, out of which only 4% of the alveolar surface is covered by AEC-II and the remaining 96% is captured by AEC-I. The nucleus of AEC I is tiny and excessively thin, curtailing the diffusion interval between the pulmonary capillary blood and alveolar air space [31]. In addition, by secreting pulmonary surfactant, the alveoli’s surface tension falls [32]. In response to alveolar epithelium damage, AEC II transformed into AEC I, participating in the repair of alveolar epithelium injury [33]. While lung alveolar injury may be compensated for by this mechanism, repeated exposure to very fine particles created by industrial expansion and civilization has been shown to initiate a wide variety of lung diseases [32,34]. Particularly in light of recent climate change, increasing attention needs to be paid to the public awareness of nanoparticles (NPs) and the health effects. These needs are addressed by studying the origins of NPs and their biophysical properties. NPs are also being investigated for their pathological effects on the lungs at the cellular level, as well as how they interact with biological defense systems [35]. In terms of their physicochemical properties, nanoparticles characteristics, such as dimension, arrangement, charge, molecular and functional form, crystallinity, surface area, solubility, and surface derivatives, are thought to be the determinants of toxicity (Table 1). Consequently, this physical property of NPs permits the interplay between inflammatory mediators alongside enzymes or proteins in cells. Therefore, it aggravates injury and inflammation [36], explaining why nanoparticles are less beneficial to cells and tissues. It is interesting to note that lung structure influences the toxic effects of NPs. The actual targeting of molecules in the upper and lower respiratory systems depends on the clearance mechanisms of the respiratory tract and the existence of cellular layers that require crossing. In contrast to the upper respiratory airways where the bronchioles and bronchi are covered with a mucus lining/coating, inside the lung alveoli, there is only a single-cell layer that separates inspired air mist from blood capillaries. This concept is very crucial for the development of particulate drug delivery, especially targeting pulmonary vasculature in PAH [37,38]. Additionally, the targeting of pulmonary vasculature could also be facilitated by taking advantage of the largest surface area of AEC I. During the progression of PAH, AEC II is exposed to hyperplastic proliferation to shield the uncovered basement membrane, possibly due to mechanical injury to AEC I [31]. In addition, AECII in the alveolar cavity undergoes apoptosis and differentiation to form AEC I for the replacement of the alveolar surface [39,40]. Eventually, a repair network formed by distal bronchial epithelial cells in response to inflammation, including club cells, lymphocytes, pericytes, alveolar macrophages (AMs), and endothelial cells, in PAH is essential for maintaining cellular homeostasis, all of these can be mechanized as bio-triggered mechanisms for drug release and are targeted in PAH [41]. Resident (myo) fibroblasts generate a collagen-rich extracellular matrix for the restoration of the damaged alveolar surface during the progression of PAH. The accumulation of inflammatory mediators and cellular debris in the lung alveoli can further amplify vascular remolding. Additionally, the use of novel particulate drug deliveries may sometimes lead to physicochemical burdens on epithelial surfaces. For example, certain shapes of particles may have cytotoxic potential [42]. Furthermore, the solubility and retention of inhaled particles must also be considered, as they can easily accumulate within biological systems due to low solubility or degradation and persist there for a longer period. Therefore, certain characteristics of particulate drug delivery not only influence the efficacy of molecules but also have potential detrimental effects on pulmonary vasculature. Targeted drug delivery to the pulmonary vasculature may cause inflammatory responses because of its distinctive physiochemical properties. The subsequent commencement of innate immunity as a first line defensive response along with macrophages helps remove drug particles. The inhalation of drug particles for targeting vascular remolding in PAH also triggers an influx of alveolar macrophages (AMs) [43]. AMs are recruited to injury areas, which further leads to the accumulation of macrophages and expression of various types of cytokines, chemotaxins and other immune cells [44]. The series of sequences leads to the generation of numerous cytokines Viz. IL-1 and TNF-α, which exacerbates immune responses at pulmonary vascular endothelial cell surfaces and epithelial lining, which assembles pro-inflammatory mediators, thereby mobilizing leukocytes [35]. Infiltration of inhalation particles into airways may exacerbate the already present reactive oxygen species (ROS) level. During progression, vascular remolding increases the number of neutrophils in the body, one of the main types of leucocytes. Neutrophils are likely to be the first type of leucocyte found due to the infiltration of inhalation particle in airways [45]. Therefore, particulate inhalation-dependent inflammatory responses are a result of the continuous flushing of ROS levels. ROS is produced in our bodies by aerobic cells through cellular respiration [43]. All these events contribute to the formation of plexiform lesions, a marker of severe PAH, which are complex vascular formations characterized by disordered angiogenesis, thrombosis, and inflammation [46,47]. Singlet oxygen [^1^O_2_], superoxide [O_2_^−^], and hydroxyl [HO•] radicals deactivate endothelium-derived nitric oxide (NO) and, therefore, boost the advancement of vascular endothelial dysfunction owing to their high reactivity and destructiveness [43]. The inhibition of PDE5 increases cGMP levels and NO production in the lungs [48]. The details of novel targets, their corresponding molecules, and the available formulation irrespective of inhalation route are shown in Figure 3 and Table 1, respectively. Consequently, increasing NO levels in the tissues reduces pulmonary vascular resistance and may benefit patients with pulmonary hypertension. As a powerful pro-fibrotic growth factor, TGF-β increases the expression of collagen extracellular matrix, promoting lung fibrosis by enhancing its expression [49]. Gold nanoparticles of a broader size range (2–200 nm primary diameter) accumulate in the blood and liver following the inhalation of mice, with the translocation of particles less than 10 nm demonstrating the greatest accumulation [50] (Table 1). A direct link between cardiovascular disease and nanoparticles in the ambience can be accounted for by the accumulation of inhaled nanoparticles in systemic circulation and in places of vascular impairment. Studies have also found that the size of inhaled particles plays a key role in determining the sequestration of these particles by immune cells and their clearance from the blood flow [51]. Based on the results, a conclusion can be drawn that repeated exposures to nano-sized particles and nanometal-based particles may have harmful health effects. In vitro studies have revealed that particles with diameters greater than 200 nm prefer to interact with tissue-resident APCs, whereas minor particles (200 nm) can be mobilized by lymphatic drainage or venous circulation and taken up by tissue-resident dendritic cells (DCs) [52]. The analysis strongly suggest that the type of interaction depends on the size of nanoparticles in alveolar-vascular tissues. Immune responses frequently rely on identifying antigens found in microbial cell proteins or pathogen-associated molecular patterns (PAMPS) [53]. Moreover, the identification of self-derived molecules released by deteriorated and necrotic cells is a crucial aspect of innate immunity by the danger-associated molecular pattern (DAMP) receptor, which is prominent for the initiation of an immune response without any resistance in response to NPs [54,55]. Adaptive immunity takes numerous days to be functional and is facilitated by helper T cells, which are the most important for immune responses. In contrast, innate immunity is available instantly. When NPs enter the bloodstream, they interact with red blood cells and white blood cells and complement proteins and other components of the blood. TH1 effector cells are differentiated from APCs such as dendritic cells, which activate naive helper T (CD4+) cells, and both cell-surface B7 and secreted IL-12 [56]. Therefore, bacteria that are sheltered within the macrophages’ phagosomes were killed by macrophages after activation by interferon, TNF, and interleukin (IL)-2, which are initialized and expressed by TH1 effector cells [57].

As a result of its CD40 ligand and secreted interferon, a TH1 effector cell triggers macrophage activation, contributing to the killing of intracellular bacteria [72]. By activating T cells, cytokines released from a TH1 effector cell cause active T cells to bind to the MHC-antigen complex expressed on infected cells. Activated T cells then differentiate into cytotoxic T cells (CD8+ T cells), which then lyse infected cells. When a TH2 cell is activated, B cells produce most classes of antibodies, including antibodies that bind to basophils, eosinophils, and mast cells [72]. The adaptive immune response is also triggered by ultra-fine particles. However, unlike bacteria and virus infections, such small particles may interfere with cellular or molecular functions since they are not composed of proteins, regardless of the attached proteins through their bio-corona [73]. The most common cell type to process NPs is the DC [74].

A human-made material, including nanomaterials, can actuate DCs specifically or by implication through tissue harm. Corona-coated nanoparticles or protein aggregates are termed nanoparticle-associated molecular patterns (NAMPs) and bind to the pattern recognition receptors (PRRs) on DCs [74], activating a danger signaling cascade that initiates DC maturation. ROS, cytokines, chemokines, and co-stimulating molecules successively released by DCs will activate naive T cells and activate inflammasomes, which increases caspase-1 activation and promotes pro-IL-1* synthesis. The NP size measurement can influence the development of T cell subtypes in the same way that its shape predisposes to specific routes of uptake into cells as well as more proficient uptake into various cell types [75]. Ikeda et al. (2010) demonstrated that the stimulation of platelet-derived growth factor (PDGF) increases the growth rate of PASMCs, which is vital to the pathogenesis of PAH [76]. The details of pre-existing formulations of the molecules that are already reported to have potential in the treatment of PAH were presented regardless of the inhalation route. The details shown in Table 1 might be helpful in predicting the competency of molecules to be fabricated via inhalation or aerosolization. 

## 4. Particle Deposition at Pulmonary vasculature and Inhaled performance

Drugs deposited in the peripheral airways are cleared rapidly, resulting in a short duration of action [77]. Additionally, the inhalation route for targeting PAH has many challenges, such as the ability to provoke an immune response, inaccurate dosing, and instability [78,79].

### 4.1. Duration of action

A major advantage of pulmonary administration is rapid drug absorption. This may result in multiple daily dosing due to the moderately brief length of clinical impacts [80]. In order to address this issue, various approaches have been implemented, some of them utilizing biodegradable polymers and microcrystalline protein molecules. The research was undertaken to see if it was possible to load medications into poly-lactic glycolic acid (PLGA) microspheres for aspiratory conveyance [77,81]. The study found that the sustained release of drug was possible with pulmonary delivery with certain improvements to the processing/manufacturing conditions for the drug loaded PLGA microspheres. Furthermore, the PEGylating of proteins has been utilized to achieve prolonged systemic action via the lungs. [82,83]. Other benefits of proteins coupled to PEG include greater bioavailability, longer plasma half-lives, reduced immunogenicity, reduced proteolysis, and improved solubility and stability. [84,85]. The primary issues with biodegradable polymers are the deposition of the polymer in the lungs and the loss of protein activity during the production of microspheres. [86,87].

The aerosolisation of proteins and peptides can reduce their biological activity. Polymers such as PEG are useful for maintaining the chemical stability of proteins after formulation [88]. The mucosal surface and lung cell lines may suffer from antigenic encounters by aerosol inhalation [89] of certain drug-like proteins, which is possibly indorsed by parenteral immunization, resulting in local, pulmonary cell-mediated immunity. Additionally, the probable immunogenic response after inhalation of aerosol mist would be reduced after coupling PEG to a protein, as demonstrated by [90]. Another method has been to use sugars such as lactose. 

### 4.2. Scarcity of Dosing Accuracy

Despite monotherapy, many PAH patients fail to meet their treatment goals. Additionally, a risk of high-output cardiac failure can be caused by excessive dosing [91]. Multidrug treatments are appealing since each of the FDA-approved PAH medication classes works by a different mechanism. Two or more drugs can create an enhanced effect or may minimize adverse effects by allowing dosing at levels below those that cause adverse effects [92]. The specific devices that are an important part of pulmonary administration may pose diverse challenges to transporting large amounts of drugs to pulmonary capillaries. Human scintigraphy data with inhaler devices advocate a little certainty about the amount of medication reaching the target site in the lungs [9]. The reason behind this is found to be associated with the amount of drug that is left behind in the spacer system or/and devices. Additionally, the aerosol mist generated through these devices mainly impacts the portion of the throat from where it is ingested into the esophagus via the laryngopharynx [79]. Sometimes, dose-dependent adverse effects of a particular drug may also limit its use. For example, subcutaneous Treprostinil might not be safe at high doses if there is frequent, severe pain at the injection site. As a result, Treprostinil may not be as effective as advertised owing to its dose-dependent effect on walking distance over a 6-min period [93].

### 4.3. Aerosolized Particle Size

A key to effective dealing with pulmonary arterial hypertension is to target alveoli in view of the lesion site. Furthermore, it also aids in reducing extra pulmonary side effects [94]. The two important parameters that predicts the deposition site of aerosol particles are (mass median aerodynamic diameter) MMAD and (Fine Particle Fraction) FPF. MMAD is an important parameter that is used to unfold the diameter of particles generated from aerosol mist. In brief, the particle size distribution of half of the generated aerosol mist denotes the aerodynamic diameter of particles [95]. The preferred MMAD for targeting pulmonary vascular lesions at alveoli is between 1 and 3 μm [80]. Numerous reports are in agreement with this particle size distribution to infuse alveolar-capillary epithelium for treatment of PAH. In particular, particles having an aerodynamic diameter of ≤2.5 μm are able to infiltrate all the way down to dense alveoli. This aerodynamic mist may comprise a suspension of liquid and solid droplets or particles [96] (Table 1). Therefore, particle size influences the targeting capacity of the formulation for treating vascular remolding in pulmonary and cardiovascular lesions.

Various forces that collide on inhaled aerosol mist fluctuate constantly during the phases of inhalation and exhalation. In particular, for larger particles possessing a diameter of more than 5 µm, inertial forces display a significant impact to the withdrawal of particles from aerosol before entering the windpipe. The events that occur during inhalation are crucial when aerosol progressively gears up for peak inspiratory flow rate. As a result, the larger particles could just hit the oropharyngeal area or upper airways and be eliminated from the nose, mouth, or throat. When aerosol progresses towards the bronchi, the speed of aerosol mist slows down and particles having an aerodynamic diameter of 1–5 µm start to sediment via gravity. Furthermore, increased cross-sectional area at lower airways aids gravitational sedimentation. The slower rate of the passage of aerosol particles from bronchi to clusters of alveoli ultimately leads to a prolonged residence time. Additionally, the denser alveoli cluster further aids gravitational sedimentation. At this point, for particles having a diameter of less than 3 µm, Brownian motion comes into existence to increase their accumulation in the pulmonary region. The pivotal rationale could be deaccelerated air in this instance and progressive lessening of the diameter of alveoli’s (average size of the alveolus is 200 µm [25]). Furthermore, the cohesive drive can be more noticeable for smaller particles than for larger particles; as a result, smaller particles would require a higher flow rate to deagglomerate. A narrow particle size distribution, on the other hand, disperses more quickly, but the impact is less than a broader particle size range [97,98].

The respiratory system can also capture particles that are not deposited during inhalation, and rapid changes in airflow direction (from inhalation to exhalation) may intensify particle deposition [99]. Therefore, targeting the alveolar-capillary epithelium in PAH is mainly dependent on particle size. Various computation models and in vivo studies place emphasis on particle size modulation for the efficient targeting of molecules [100]. The liberal loss of particles in due course of the exhalation process ultimately leads to a reduction in particle deposition frequencies. To estimate how many particles of various sizes should be deposited in specific lung regions, one must consider breath dynamics and particle parameters such as density, shape, and individualized respiratory tract geometry [101,102]. Nevertheless, even though particles with extremely small diameters such as nanoparticles have more potential for deposition in the lesions of pulmonary vasculature, they are also prone to being expelled from generated aerosol mist by inhalation devices [103,104,105]. The sudden deliquescence tendency of particles in mist may cause the size of aerosol particles to grow due to coagulation/coalescence and hygroscopic growth; aerosol droplets produced in the breathing phases are also prone to elimination via exhalation [106,107]. As a result, the viable option seems to envelop the medicine in particles with mass densities < 0.4 g/cm^3^ and geometric diameter > 5 mm, which may improve respiratory deposition, as illustrated by Edwards et al. [108] Varying the polymer concentrations and methods of production of particles largely influenced the final size of aerodynamic mist. Therefore, optimization and particle engineering are crucial before the introduction of particles into inhalation devices.

#### 4.3.1. Mass Median Aerodynamic Diameter

The mass and buoyancy of a spherical particle that is equal to a randomly shaped airborne particle are represented by MMAD. The interrelation between polymer physicochemical properties and the final aerodynamic size could be put forward with theoretical MMAD and actual MMADa [109,110]. The former could be determined from tapped density and volume-based mean diameter, while the latter was calculated from the Anderson Cascade Impactor. Both are determinants of particle deposition patterns via inhaler devices and, consequently, lung deposition. Impaction, sedimentation, and diffusion are the three principal deposition patterns that operate on all entrained particles; nevertheless, each mechanism impacts certain sized particles inside a specific airway area. The efficient deposition of drug molecule to the respiratory region, especially to the lower portion of the lungs, is represented by MMAD with < 3 µm. Formulations having larger particle sizes are filtered initially in nasal passage (MMAD ≥ 5 µm). Particles with very fine size of <0.5 µm MMAD may reach the lung parenchyma through an inspired airstream. The deposition behavior of particle in aerosol process is established by particle MMAD. To calculate the actual MMAD of formulations, the weight of formulations deposited on each plate is measured [111]. The impacted flow rate in L/min should be associated with the inhaler device used for the simulation of aerosol mist [112]. The following formula was used to compute the theoretical mass median aerodynamic diameter (MMADt) of the product. It was also calculated from tapped density measurements by using the following equation:

(1) $$\mathrm{MMADt}=\delta {\left(\frac{\rho }{\rho_{0}X}\right)}^{1/2}$$

where *δ* is the geometric mean diameter calculated from particle size analysis, ρ is the tapped density, ρ_0_ is the reference density of 1 g/cm^3^, and “*X*” is the shape factor, which is 1 for a sphere.

#### 4.3.2. Fine Particle Fraction

The fraction of the drug mass included in an aerosol mist that is small enough to reach the lungs and exhibit a therapeutic impact is known as fine particle fraction (FPF). It is mostly used to assess the quality of aerosols. FPF is described in terms of the amount of medicine recovered from distinct impactor stages; however, the precise definition in terms of a particle size range varies depending on the impactor type. The mass percentage of released particles less than 5 μm in relation to the recovered dose is also one of the critical parameters, especially for Dry Powder Inhaler (DPI) formulation [113].

(2) $$\mathrm{Emitted}\;\mathrm{Dose}\;\left(ED\right)\%=\frac{\left(\mathrm{Initial mass in capsule}\right)-\left(\begin{array}{c} final\;mass\;remaining\;\\ in\;capsule \end{array}\right)}{\begin{array}{c} \mathrm{Initial mass}\\ \mathrm{in Capsule} \end{array}}\times 100$$

(3) $$\mathrm{FPF}\;\left(\%\right)=\frac{\mathrm{Mass}\;\mathrm{of}\;\mathrm{the}\;\mathrm{particles}\;\mathrm{in}\;\mathrm{stages}\;0\;\mathrm{through}\;5\;\left(\mathrm{of}\;\mathrm{Cacade}\;\mathrm{Impactor}\right)}{\mathrm{Emitted}\;\mathrm{dose}\;}\times 100$$

The fine particle fraction (FPF) is the ratio of fine particles with an aerodynamic diameter of 5 μm emitted to the dose emitted of 100 [114]. In other words, after every actuation on the impactor, FPF is evaluated by dividing the number of drugs deposited on the impactor (stage 6) by the amount of drugs recovered per capsule [115]. A higher fine particle fraction value indicates deeper lung deposition. The emitted dose was determined by dividing the dose emitted from the inhaler device by the total dose [116]. Emitted dose (ED) is the fraction of powder that vents out of the device after actuation, where the fraction is equivalent to the mass of powder recovered in impactor stages, attributed to the mass of powder collected on all impactor stages, induction port, inhaler device, and the capsule together. The contents of deposited drugs were collected and assessed by suitable analytical methods such as HPLC.

### 4.4. Devices for Inhaled Administration

**Figure 4:**
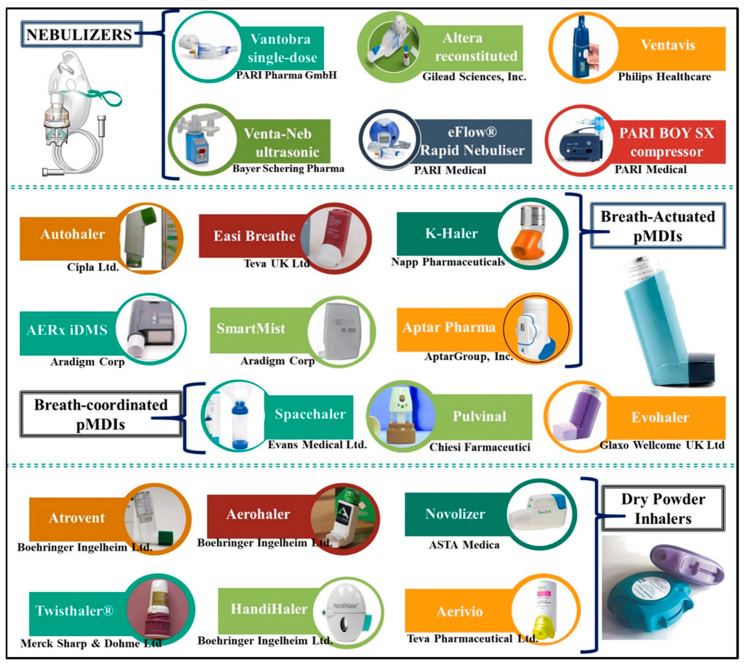
Representative example of devices (brand names) for inhalation drug delivery.

Inhaled medications have certain advantages in PAH. Those who consume inhaled medication through a nebulizer, MDI, or DPI report less flushing and diarrhea. Below are a few of the important inhaled medications that are approved for treating PAH. Figure 4 illustrates the inhaler devices that could be used for the delivery of medication through aerosolized mist.

#### 4.4.1. Nebulizers

A nebulizer is a machine that creates an aerosol from a liquid or solid vapor solution. Inhalational nebulizers deliver drugs to the lower respiratory tract. Insufflation devices and formulations contribute to pulmonary bioavailability in significant ways. Nebulizers have been used to treat a wide range of lower respiratory tract illnesses for decades.

##### Jet Nebulizers

A mist of aerosol particles is produced when a compartment containing the liquid formulation is activated. In these nebulizers, a corrugated tube serves as a reservoir, which creates an aerosol when inhaled. Aerosol formation results in a significant amount of medication loss [117]. A breath-enhanced jet nebulizer helps the patient breathe while inhaling; hence, they are called breath-enhanced jet nebulizers [118]. Nebulizers that produce aerosol when the patient inhales are called breath-activated jet nebulizers [119]. This method reduces medication loss, but it has lengthened the treatment period.

##### Ultrasonic Nebulizers

High frequency ultrasonic vibrations are passed through the formulation to create the aerosol. Ultrasonic nebulizer relies on a piezoelectric transducer to generate high-frequency vibrations of 1–3 MHz using electrical impulses rather than compressed air or gas [120].

##### Mesh Nebulizers

Vibrating mesh nebulizers driven by battery or electricity using micropump technology were established to overcome the limitation associated with jet and ultrasonic nebulizers. A nebulizer consists of a mesh with micron-sized numerous holes that generates an aerosol by vibration [121].

#### 4.4.2. Pressurized Metered Dose Inhalers (pMDIs)

A pressurized inhaler that uses propellant sprays to deliver medicine to lungs is a metered-dose inhaler (MDI). These are most often utilized medication delivery devices for asthma and COPD patients [122].

#### 4.4.3. Dry Powder Inhalers (DPIs)

DPIs (dry powder inhalers) are non-propellant portable devices. DPIs alleviate the fundamental limitation of MDIs as it requires little patient coordination between breathing and aerosol inhalation [123].

#### 4.4.4. Iloprost (Ventavis)

It is one of the oldest medications that has been marketed as an inhaled medication. It is a prostanoid called Ventavis that was approved in April 2005. Ventavis is used for almost all types of pulmonary arterial hypertension. Ventavis is an inhalation medicine administered using a nebulizer. It is recommended to take 6–9 doses a day [124,125].

#### 4.4.5. Treprostinil (Tyvaso)

Tyvaso was authorized for use in PAH patients with moderate to severe symptoms and who wanted to increase their walking distance since July 2009. Tyvaso plus an ultrasonic nebulizer and accessories were authorized as a drug–device combination product. The patient should receive four doses per day at first, while increasing the number of breaths per dose as the therapy is optimized. Administering the drug requires the use of a nebulizer machine that is unique and personalized [126].

## 5. Novel Formulation Approaches

### 5.1. Lipid Nanoparticles

Lipids are one important group of polymers that may provide protection against undesirable immune responses via pulmonary administration [117,118]. Liposomes, SLN, NLC, and micelles are core lipid formulations that may be useful for active deposition and the extension of drug release in pulmonary vasculature. Therefore, lipid bilayer structure vehicles such as liposomes have better tolerability; they might be a promising candidate for drug delivery with reduced dosing frequency and less extra pulmonary drug deposition, specifically in pulmonary arterial hypertension [119]. Furthermore, suspension or colloidal dispersion can be effectively delivered via nebulization. Different methods, such as spray drying, spray freeze drying, and freeze-drying, were effectively utilized to produce liposomes [127]. However, despite several advantages, the reconstitution of suspension from dry powder liposomal preparation is a tortuous route for the formulator. Furthermore, the distribution of particles sizes in liposomal dispersion and stability in relation to the withholding capacity of liposomes is also questionable. Liposomal preparations have been utilized to load a wide range of medicaments and are used to treat many ailments [128]. Currently, Amikacin Liposomal inhalation suspension already approved by the United States of America as a classic example of an extended-release formulation for pulmonary delivery, which is utilized as a portion of the combination for an anti-bacterial dosage regimen for Myobacterium avium complex in lung disease [129]. Amikacin was enclosed in a lipid bilayer made up of dipalmitoylphosphatidylcholine (DPPC), an exemplary excipient utilized in inhaled liposomal formulations [130]) and cholesterol. For a blend of two drugs, liposomes have also been tested for the treatment of pulmonary arterial hypertension. Fasudil in combination with diethylenetriamine NONOate collectively decreases the mean arterial pressure [131]. The liposomal formulations consist of both drugs separately encapsulated in unmodified and cyclic peptide (CARSKNKDC) CARs modified liposomes [132]. In the acute study (monocrotaline-induced pulmonary arterial hypertension in rats) MCT model, monocrotaline is a pyrrolizidine alkaloid excavated from the plant Crotalaria spectabilis. The enzyme, cytochrome P450 3A, stimulates monocrotaline in the liver to result in the formation of toxic metabolites 6,7-dihydro-7-hydroxy-1-hydroxymethyl-2-pyrrolidine (DHP) and dehydromonocrotaline (DHM). These toxic metabolic products are expected to damage pulmonary artery endothelial cells, initiating complex pathological alterations that mimic several key aspects of pulmonary hypertension. Some studies with liposomal formulations showed the fast absorption of drugs in MCT models through the entrapment of mist, an important corollary to extreme vasodilation in the pulmonary endothelial vasculatures. This might lead to rapid reductions in mean pulmonary arterial pressure (mPAP) [133,134]. Chronic studies advise that liposomal formulations are capable of reversing collagen deposition. Additionally, a study with the SUGEN5415/hypoxia model decreases mPAP equally because it improvises pathological features and stabilizes several biochemical and cellular fluctuations that occur in the pulmonary vasculature [135]. In a similar fashion, fasudil and the enzyme Superoxide dismutase (SOD) were also incorporated into liposomes by using a freeze–thaw method. The liposomes were developed by solvent evaporation, thin-film formation, and hydration techniques to generate empty unilamellar vesicles. They reported efficient uptake of the formulation into endothelial and smooth muscle cells.

Similar to 1,2-Distearoyl-sn-glycero-3-phosphocholine (DSPC), lipids could also be tailored with dipalmitoylphosphatidylcholine, especially in the case of pulmonary arterial hypertension. Dipalmitoylphosphatidylcholine (DPPC) lipid nanoparticles (DPPC-LN) has been reported for Resveratrol for favorable therapeutics for pulmonary arterial hypertension [136]. These particles were efficiently formulated with a thin film hydration-ultrasonic dispersion technique using glyceryl monostearate as the lipid core. DPPC to prevent lipid nanoparticles from colliding with pulmonary surfactant. DPPC-LN are preferred in this regard due to their greater potential for drug localization at the pulmonary vascular wall and prologue residence time of drugs in the lungs.

#### 5.1.1. Solid Lipid Nanoparticles

Another more stable formulation is SLNs due to their high morphological rigidity and encapsulation efficiency. However, they may increase pulmonary burden when offered for sustained release medicament due to faster degradation [129]. They can be easily developed via different techniques such as solvent-diffusion and modified melt emulsification technique. SLN PDI, zeta potential, percent drug entrapped, and particle size (Table 1) were measured using photon correlation spectroscopy (PCS), Zeta Sizer Nano-ZS90 (Malvern Instrument Ltd., Worcs, England) and other instruments [130,131,132].

A recent study by A. Liparulo et al. effectively delivered 3-dodecyl-4,5-dimethoxy-1,2-benzoquinone via SLN formulation [137]. Additionally, they found that SLN showed a controlled release of the above medicament at PAH sites with significant reduction in mean pulmonary arterial pressure. However, the morphological studies show that SLNs have specific crystallized structure, which may attribute to drug loading capacity [133]. The study utilizes hydrogenated coconut-glycerides as a suitable lipid matrix in SLN preparation due to their characteristics low melting point (35 °C), which have capability to bear thermal stress conditions during formulations [134,135].

#### 5.1.2. Nanostructured Lipid carriers

NLCs are biodegradable, potentially safe, and able to extend medicament exposure to the pulmonary site. Additionally, despite its smaller structure, more drugs are accommodated in NLC compared to SLN. Although NLC formulations are newer and have some edge over SLN’s, the characterization techniques of NLC and SLN are similar [136]. At its core, it is made up of solid–lipids such as precirol, compritol, or beeswax and liquid–lipids such as oleic acid and capmul MCM. NLCs can be manufactured via various techniques such as solvent-emulsification evaporation, solvent-emulsification diffusion, double emulsion, and membrane contactor [138]. Ethyl pyruvate is also encapsulated in nanoparticles formulated by the emulsion solvent-evaporation method to retain the drug in lung tissues. Study reports show that intratracheal instillation not only inverts the remodeling of the arteries but also blocks the generation of proinflammatory factors, consequently reducing pulmonary circulation resistance [139].

### 5.2. The Micelles

The micelles are self-assembled systems with hydrophilic and hydrophobic properties, often used for inhalational therapy of pulmonary arterial hypertension. They can escape attacks by alveolar macrophages due to their small size and massive hydrophilic shell. Therefore, micelles can be tailored for better biocompatibility and prolonged residence time as polymer–lipid conjugates, such as PEG, PEGylated lipid, and PVA [140,141]. While micelles based upon block copolymers may degrade slowly, the chances of an increase in pulmonary load may be higher [142,143]. Phospholipids and amphiphilic molecules are also used to prepare micelles for enhanced physical stability [144,145] and higher residence time in the lungs. One of the major excipients during the formulation of micelles for pulmonary administration of drugs is PEG–DSPE by virtue of their protracted fatty acyl sequences, which form a drug entrapped aureole. The PEG–DSPE micelles are able to seize a good quantity of medicament, which ensures the optimum drug release within the time frame of the therapeutics index [146]. Additionally, PEG–DSPE micelles are able to be efficiently nebulized due to their small size and deeply hydrated shell; thus, they are proficient at escaping AMs. The solvent evaporation method could be used to manufacture poly (ethylene oxide)-block–distearoyl phosphatdylethanolamine (PEG5000–DSPE) micelles [147,148].

Micelles are characterized by micelle size determination, zeta-potential measurement, and stability (Tablet 1). Another approach for poor water-soluble drugs for targeting pulmonary arterial hypertension is via aqueous suspension of pluronic^®^-mixed micelles (PMM) [11]. The particles could be generated with a thin-film hydration technique. Hu, X. et al. proposed the application of PEG–PLGA micelles for the treatment of PAH for the ester prodrug curcumin acetate [149,150]. They found that PEG–PLGA particles were efficiently received by alveoli, which were cuddled by blood capillaries brought by the pulmonary vascular endothelium. Duy Toan et al. provided a detailed classification of polymeric micelles [151].

### 5.3. Particle Size and Lung Retention

The most practical and effective method to modify the physicochemical properties of nanoparticles is to modify their size, and efforts have been made to increase their lung retention duration by modifying their size. Kreyling et al., and Andersion et al., studied the effect of particle size in relation to its proportional positive or negative influence on lung retention [152,153]. The particle size of SLN, which is less than 1 m, may be able to evade macrophage phagocytosis. However, several studies have shown that lipid-based nanoparticles with diameters of 100–300 nm could be phagocytes. Furthermore, the particle size had an inverse relationship with clearance rate. Particles that enter cilia are more likely to be ultrafine [154].

## 6. Conclusions

In view of the adverse functional changes associated with PAH and related pathological variability, there is a myriad of studies indicating newer molecular therapies for PAH. It is, therefore, imperative to examine molecular mechanisms that underlie the pathophysiology of PAH in order to develop newer therapeutics and drug delivery systems. However, the correlation between inhaled formulation and pre-existing drug delivery should be established. Targeting drug delivery in PAH will certainly require both conceptual and technological advances [113]. The development of advanced inhalation platforms will result in the highly effective delivery of drugs to a specific site of PAH. The performance and effectiveness of newer formulations are influenced by the mechanism involved in the pathologies of PAH and its detrimental effects on inhaled drugs. Therefore, many efforts are required to delve into the understanding of rigorous formulation parameters and their correlation with crucial inhalation targeting newer therapeutics.

Although a great deal of progress has been made in our understanding of the pathophysiology of PAH and the discovery of new therapeutic agents, clinical efficacy via aerosolized medication encounters a major challenge in patients with PAH. There are several reasons for these challenges, including the inability to optimize the effectiveness of therapy and the lack of an accurate monitoring of the drug’s effects in reversing the underlying pathological vascular remodeling of arterioles in the lungs. Treprostinil, palmitil, rosiglitazone, fasudil, superoxide dismutase (SOD), prostaglandin E1, nitrite and nitrate, erlotinib, ethyl pyruvate, bosentan, and many more are examples [57,69,86,87,138]. Additionally, recent investigations into implantable devices have been introduced for the treatment of pulmonary arterial hypertension. The principal safeguard mechanism of the pulmonary tract against any mechanical declamation includes beating cilia, macrophages, and mucus, which majorly influence the deposition of inhaled particles [32]. Additionally, aerosols targeted at the lungs must be designed so that the maximum aerosol diameter is no larger than 5 µm. In vitro studies related to the micromeritical parameters of inhalation delivery should be correlated with the actual site of deposition and consequent efficacy to define potential outcomes. Furthermore, reported laboratory studies in animal models of pulmonary hypertension on the existing formulation would reciprocate its clinical effectiveness in stimulating controlled vasodilation solitary in pulmonary arteries. Currently, the available therapeutics for PAH are ineffective due to a lack of critical understanding of its pathophysiology. It is noteworthy to mention that inhaled formulations or delivery systems suffer from a widespread limitation owing to their short duration of action and inspiratory flow pattern [50]. A major cause of the pervasiveness of inhaled deposition to PAH is the structural differences in the alveolar and epithelial barriers [154]. Additionally, the metabolic instability of the alveolar epithelium may also aggravate these limitations.

The complexity of the cardiopulmonary microenvironment makes drug delivery to the alveolar–capillary region more difficult. Furthermore, in vitro data from the intratracheal instillation of drugs and its correlation with in vivo performance are questionable. Although existing animal models and in silico studies have been proven to have some edge in predicting clinical performance, drug deposition to the infinitesimal smaller portion of pulmonary vasculature is suspicious. In our view, in addition to discovering molecular targets via inhaled drug deposition, the molecular site is crucial for achieving the desired therapeutic effect. We also believe that targeted pulmonary drug delivery is not only restricted to the lung as a whole; rather, of the approach should be to deliver medication more and more close to the pathological nanoscopic sites. We believe, in addition, that the noticeable inferences include the finer optimization of drug delivery at micro-molecular regions, the faster achievement of drug localization, and adequate therapeutic responses.

## 7. Future Perspective

Targeting drug delivery in PAH will certainly require both conceptual and technological advances. The development of advanced inhalation platforms will result in the highly effective delivery of drugs to a specific site of PAH. The performance and effectiveness of newer formulations are influenced by the mechanism involved in the pathologies of PAH and its detrimental effects on inhaled drugs. Therefore, many efforts are required to delve into the understanding of rigorous formulation parameters and their correlation with crucial inhalation targeting newer therapeutics.

Pharmacotherapies for PAH are hampered by their short half-lives and instability, which result in decreased efficacy and an increased risk of systemic side effects. While research in this field has increased, there is still no cure for PAH. The pneumonic course has been proffered to overcome the inadequacies of anti-PAH drugs. Therapeutics that are nano-entrapped in nebulized form may well be utilized for the treatment of pulmonary hypertension with better bioavailability and fewer side effects. In order to make strides, the adequacy of ordinarily endorsed and investigational drugs focused on conveyance has been investigated by an analyst. The world view shifts within the pathogenesis of pulmonary hypertension from primarily vasodilatory to remodeling methodologies tending to the ailing aspiratory supply routes with cancer-like hyperproliferative highlights, opening up unused roads for innovative treatments. The surface alteration of these carriers with a target specific component that binds to receptors found on pulmonary smooth muscle cells or endothelial cells might also enhance the medicine’s specificity. The strategies that are defined by selective and specific targeting of pulmonary circulation with minimal extra-pulmonary side effects and reduced dosage and administration frequency have an edge over all currently available therapies in PAH. It is important to consider, however, their safety profiles before clinical applications. Controlled release nano-formulations delivered by inhalational route can circumvent these existing limitations. A broader view is required to create secure and viable treatment options for PAH patients.

## Figures and Tables

**Table 1:** Targeting pulmonary arterial hypertension via inhalation: pathological barriers, particle deposition, and approaches.

MOLECULE	Formulation	Aerodynamic Behavior	Method of Preparation	Characteristics			References
				Zeta mV	Size (µm)	EE/LE (%)	
SILDENAFIL CITRATE	Sildenafil Citrate -loaded PLGA nanoparticles	FPF = 31.8–60.2%	Double emulsion solvent evaporation	-	4.2–18.1		[58]
SUNITINIB	Sunitinib loaded poly (3-hydroxybutyrate-co-3-hydroxyvalerate acid) nanoparticles (nps)	MMAD = 3.38 ± 0.06 μm, FPF = 61.18 ± 0.04%	Solvent-emulsification evaporation	−1.37 ± 0.24 mv	167.80 ± 0.30 nm	93.25 ± 0.03%	[59]
SORAFENIB	Sorafenib-loaded cationically modified polymeric nanoparticles	Mmad = 4 μm FPF > 80%	Solvent-evaporation	−21.5 ± 0.7 mv and −32.9 ± 2.9 mv	196.1 ± 1.1 nm (PDI 0.04 ± 0.02) and 191.4 ± 10.1 nm	38.2 ± 0.6%	[60]
RESVERATROL (RES)	Novel spray dried inhalation powder	MMAD = 3.86 ± 1.04 μm FPF = 39.89 ± 1.06%	Spray drying	−1.46 ± 1.47	<5 μm	29.1 ± 2.0	[61]
RESVERATROL (RES)	Co-spray dried (Co-SD) formulations of and budesonide and resveratrol	FPF = 42.5 ± 1.7%	Spray drying	-	1 to 5 μm	-	[62]
TACROLIMUS	Dry powder inhaler	Good Mobility of aerosol	Thin film freezing	-	-	-	[63]
	Chitosan Tacrolimus PLGA-nps	Aerosol particles with good mobility	Oil/water emulsification diffusion method	+13.6	441	37.7	[64]
	Nanoparticles	Aerosol particle at inhalable range	Modification of albumin-bound technology	−34.5 ± 0.3 mv	182.1 ± 28.5	85.3 ± 4.7	[65]
	Colloidal dispersion, powder for reconstitution inhalation	46.1% of the emitted dose was in the respirable range	Ultra-rapid freezing (URF) process	-	200 to 400	-	[66]
	Tacrolimus powder for reconstitution in deionized water	MMAD = 4.06 µm GSD = 2.7 µm. FPF = 46.1%	Thin film freezing (TFF)	-	239.2	-	[67]
	Inhalable albumin nanoparticles with bound Tacrolimus	Aerosolisation maintained for ~0.12 s after actuation, 0.04 sec intervals	High-pressure homogenization technique	−34.5 ± 0.3 mv	182.1 ± 28.5	85.3%	[59]
COENZYME Q10	Microparticles	Aerosol particle at inhalable range	High-pressure homogenizer	-	-	-	[68]
	Nanosuspensions	Good aerodynamic Size	High-pressure homogenizer (HPH)	-20	100		[69]
VIP	Dry powder formulation	Stage 3 (3.3–4.7 μm) aerodynamic size less than 10 μm,	Milled with an A-O JET MILL	-	4.5 μm	-	[70]
CARSKNKDC (CAR) *	Liposomes	That energy produced by the microsprayer did not affect liposomal Integrity	Thin-film formation, hydration, and extrusion method		152.7 ± 2.38 nm	54.91 ± 1.66 (superoxide Dismutase) 39.22 ± 3.41 (Fasudil)	[71]

* CAR is a peptide to be attached to liposomes.

## Data Availability

Majority of the articles referred are cited appropriately in the manuscript.

## Abbreviations

1,2-Distearoyl-sn-glycero-3-phosphoethanolamine	DSPE
1,2-dioleoyl-sn-glycero-3-phosphocholine	DOPC
1,2-Distearoyl-sn-glycero-3-phosphocholine	DSPC
alveolar epithelial type I	AEC I
alveolar epithelial type II	AEC II
alveolar macrophages	AMs
AMP kinase	AMPK
Bone morphogenetic protein receptor	BMPR2
Bovine serum albumin	BSA
CARSKNKDC	CAR
danger-associated molecular pattern	DAMP
dioleoylphosphatidylglycerol	DOTAP
dipalmitoylphosphatidylcholine	DPPC
Emitted dose	ED
Endothelial-mesenchymal transition	EndMT
fine particle fraction	FPF
High Mobility Group-box	HMGB1
hyaluronic acid	HA
Hydroxypropyl Methylcellulose	HPMC
Hypoxia-inducible factor 1-alpha	HIF-1α
Interleukins	IL
layer by-layer assembly	LbL
lipid nanoparticles	LN
Magnetic Nanoparticles	MNPs
mass median aerodynamic diameter	MMAD
mean pulmonary arterial pressure	mPAP
Monocrotaline model	MCT
Monocyte Chemoattractant Protein	(MCP)-1
N,N′-Dicyclohexylcarbodiimide	DCC
Nanoparticles	NPs
Nanostructured Lipid carriers	NLC
N-Hydroxysuccinimide	NHS
Nitric Oxide	NO
oxidative phosphorylation	OXPHOS
pattern recognition receptors	PRRs
PEGylated derivative of 1,2-distearoyl-sn-glycero-3-PE	DSPE-mPEG2000
Peroxisome-proliferator-activated-receptor-gamma	PPAR-γ
phosphodiesterase 5	PDE5
photon correlation spectroscopy	PCS
platelet-derived growth factor	PDGF
pluronic^®^ mixed micelles	PMM
Poly (ADP)-ribose-1	PARP-1
poly(ε-caprolactone)	PCL
polydispersity index	PDI
Polyethylene glycol	PEG
poly-lactic-co-glycolic acid	PLGA
Polyvinylpyrrolidone	PVP
Pulmonary arterial endothelial	PAECs
Pulmonary Arterial Hypertension	PAH
Pulmonary arterial smooth muscle cells	PASMCs
Pulmonary Hypertension	PH
pulmonary vascular resistance	PVR
pyruvate dehydrogenase	PDH
pyruvate dehydrogenase kinase	PDK
Reactive Oxygen Species	ROS
Right ventricle	RV
Self-Nanoemulsifying Drug Delivery System	SNEDDS
Superoxide dismutase	SOD
tissue-resident dendritic cells	DCs
Trifluoroacetic acid	TFA
tumor necrosis factor	TNF
Vasoactive intestinal peptide	VIP
